# Mechanical stretching stimulates collagen synthesis via down-regulating SO_2_/AAT1 pathway

**DOI:** 10.1038/srep21112

**Published:** 2016-02-16

**Authors:** Jia Liu, Wen Yu, Yan Liu, Selena Chen, Yaqian Huang, Xiaohui Li, Cuiping Liu, Yanqiu Zhang, Zhenzhen Li, Jie Du, Chaoshu Tang, Junbao Du, Hongfang Jin

**Affiliations:** 1 Department of Pediatrics, Peking University First Hospital, Beijing 100034, P. R. China; 2 Anzhen Hospital, Capital Medical University, Beijing 100020, P. R. China; 3 University of California, San Diego, La Jolla, California, 92093, United States of America; 4 Department of Cardiovascular Pediatrics, Capital Institute of Pediatrics, Beijing 100020, P. R. China; 5 Department of Pediatrics, Tianjin Fifth Centre Hospital, Tianjin 300450, P. R. China; 6 Department of Pediatrics, The Hospital of Shunyi District Beijing, Beijing 101300, P. R. China; 7 Key Laboratory of Remodeling-Related Cardiovascular Diseases, Ministry of Education (2014XXGB02); 8 Department of Physiology and Pathophysiology, Peking University Health Science Centre, Beijing 100191, P. R. China; 9 Key Laboratory of Molecular Cardiology, Ministry of Education, Beijing 100191, P. R. China

## Abstract

The aim of the study was to investigate the role of endogenous sulfur dioxide (SO_2_)/ aspartate aminotransferase 1 (AAT1) pathway in stretch-induced excessive collagen expression and its mechanism. The mechanical stretch downregulated SO_2_/AAT1 pathway and increased collagen I and III protein expression. Importantly, AAT1 overexpression blocked the increase in collagen I and III expression, transforming growth factor-β1 (TGF- β1) expression and phosphorylation of Smad2/3 induced by stretch, but AAT1 knockdown mimicked the increase in collagen I and III expression, TGF- β1 expression and phosphorylation of Smad2/3 induced by stretch. Mechanistically, SB431542, a TGF-β1/Smad2/3 inhibitor, eliminated excessive collagen I and III accumulation induced by AAT1 knockdown, stretch or stretch plus AAT1 knockdown. In a rat model of high pulmonary blood flow-induced pulmonary vascular collagen accumulation, AAT1 expression and SO_2_ content in lung tissues of rat were reduced in shunt rats with high pulmonary blood flow. Supplement of SO_2_ derivatives inhibited activation of TGF- β1/Smad2/3 pathway and alleviated the excessive collagen accumulation in lung tissues of shunt rats. The results suggested that deficiency of endogenous SO_2_/AAT1 pathway mediated mechanical stretch-stimulated abnormal collagen accumulation via TGF-β1/Smad2/3 pathway.

High pulmonary blood flow-induced pulmonary hypertension is one of the most common complications of left-to-right shunt heart disease with high mortality rate^1^. Along with the progress of the disease, pulmonary vascular pathological change occurs. Since the concept of vascular remodeling was first proposed in 1989, plenty of work has been done to better understand its feasible mechanisms^2^. The main pathogenesis of vascular remodeling is an imbalance between cell proliferation and apoptosis, and between extracellular matrix (ECM) synthesis and degradation^3^.

Exacerbated mechanical stretch is a characteristic of pulmonary hypertension and results in vascular remodeling. Experimental evidence showed that mechanical stretching elevated matrix metalloproteases (MMPs) expression and activity^4^,^5^, whereas tissue inhibitors of metalloproteinases (TIMPs) were reduced^4^,^6^. The enhanced ratio of MMPs to TIMPs resulted in decreased collagen degradation, which eventually caused collagen accumulation. However, the mechanism of the increased collagen expression induced by mechanical stretch has not been well-explained for.

The lung is a respiratory organ of mammals, so the sensitivity of the pulmonary circulation to gas changes is greater than that of systemic circulation. Increasing evidence showed that sulfur dioxide (SO_2_) could be endogenously generated in cardiovascular tissues in mammals^7^,^8^,^9^. *In vivo*, endogenous SO_2_ is generated during metabolism of sulfur-containing amino acids. Firstly, a sulfur-containing amino acid is metabolized to L-cysteine and then oxidized into L-cysteinesulfinate. L-cysteinesulfinate, an analogue of L-asparaginic acid, can be transaminated into β-sulfinylpyruvate under the role of aspartate aminotransferase (AAT), which is spontaneously decomposed into pyruvate and SO_2_^10^,^11^,^12^. SO_2_ is further hydrated to sulfite /bisulfite (SO_3_^2−^/HSO_3_^−^, molar ratio of 3:1) after being dissolved in water, which can be oxidized into SO_4_^2−^, and then excreted through the kidneys^13^. Therefore, AAT is a key enzyme in the generation of endogenous SO_2_ in mammals, and AAT1 is an isoenzyme of AAT which is mainly located in cytoplasm^14^,^15^. As an emerging gasotransmitter following nitric oxide (NO), carbonic oxide (CO) and hydrogen sulfide (H_2_S), SO_2_ is known for its low molecular weight, continuous generation, quick diffusion and absorbance. These properties play vital physiological and pathological roles in vascular remodeling, including inhibiting the proliferation of vascular smooth muscle cells^16^,^17^, promoting the apoptosis of vascular smooth muscle cells^18^ and reducing abnormal deposition of collagen^16^,^19^. Our previous study demonstrated that SO_2_ derivatives could alleviate the pulmonary vascular remodeling in hypoxic pulmonary hypertensive rats^20^. Furthermore, SO_2_ could decrease collagen expression in thoracic aorta in spontaneously hypertensive rats^19^.

In this regard, the present study was undertaken to examine if mechanical stretch could enhance collagen accumulation via the inhibition of the SO_2_/AAT1 pathway in pulmonary artery fibroblasts (PAFs) so as to better understand the possible mechanisms for high pulmonary blood flow-induced pulmonary vascular collagen remodeling.

## Results

### Stretch inhibited endogenous SO_2_/AAT1 pathway and increased collagen I and III expression in primary PAFs

**Figure 1:**
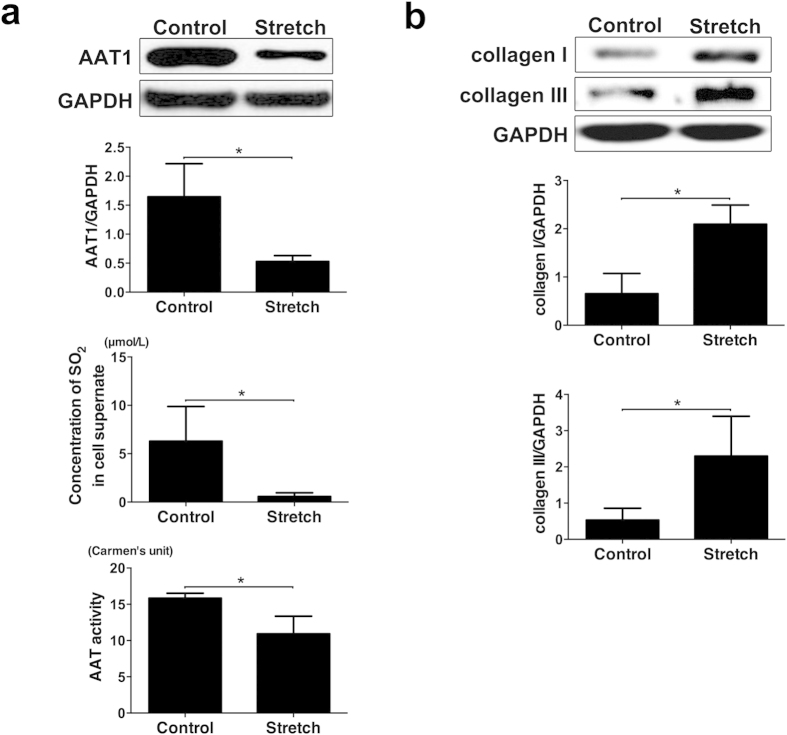
Stretch inhibited the SO_2_/AAT1 pathway and enhanced collagen remodeling in PAFs. The cells were stretched at 60 cycles/min, 1 Hz, and 20% elongation for 24 hours. (**a**) The effect of mechanical stretching on the SO_2_/AAT1 pathway. (**b**) The effect of mechanical stretching on collagen. *P < 0.05, data are presented as mean ± SD (n = 5), and two-sample independent t test was used.

To explore the effect of mechanical stretch on endogenous SO_2_/AAT1 pathway, we detected AAT1 protein and AAT activity in primary PAFs and SO_2_ content in the supernatant of primary PAFs after 24 hours of mechanical stretch. Compared with control group, the mechanical stretch significantly decreased expression of AAT1 protein and AAT activity in primary PAFs and SO_2_ concentration in cell culture supernatant of primary PAFs, respectively (*P all* < 0.05, Fig. 1a). Simultaneously, the results by western blot also showed that a 20% stretch markedly increased collagen I and collagen III expression, respectively, compared with the static cells (*P all* < 0.05; Fig. 1b). These results indicated that endogenous SO_2_/AAT1 pathway was downregulated in PAFs treated with mechanical stretch, in which collagen I and III protein was abnormally increased induced by mechanical stretch.

### Endogenous SO_2_/AAT1 pathway inhibited excessive collagen expression in primary PAFs induced by mechanical stretch

**Figure 2:**
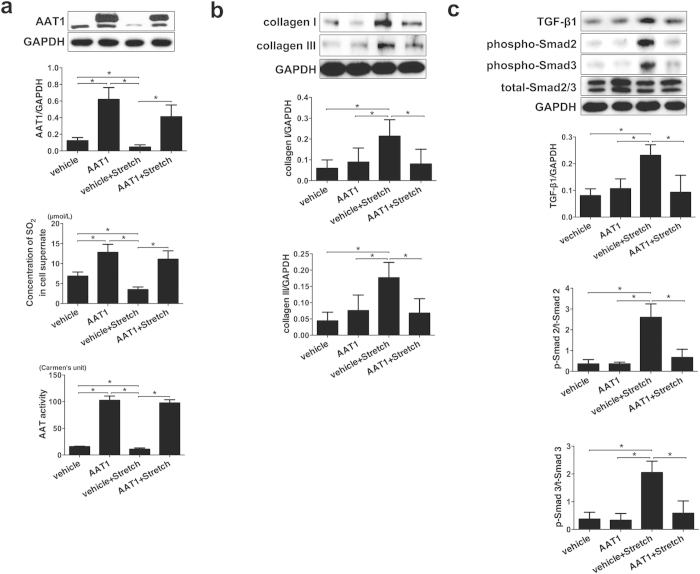
Overexpression of AAT1 prevented collagen remodeling and inhibited the activation of the TGF-β1/Smad2/3 pathway in PAFs. (**a**) The effect of AAT1 overexpression on the SO_2_/AAT1 pathway. (**b**) The effect of AAT1 overexpression on collagen remodeling. (**c**) The effect of AAT1 overexpression on the TGF-β1/Smad2/3 pathway. *P < 0.05, data are presented as mean ± SD (n = 5) and ANOVA followed by *post-hoc* analysis was used. AAT1: AAT1 overexpression.

To understand the significance of endogenous SO_2_ in the regulation of stretch-induced excessive collagen expression in the PAFs, we blocked the stretch-induced downregulation of endogenous SO_2_ levels by transfecting lentivirus vector containing cDNA encoding AAT1 into primary PAFs. Compared with vehicle group, AAT1 protein expression and AAT activity in the cells of AAT1 group were markedly increased and SO_2_ content in the supernatant of AAT1 overexpression cells was increased (*P all* < 0.05; Fig. 2a). As we expected, there was no differences in AAT1 protein expression, AAT activity and SO_2_ content between AAT1 group and AAT1 + stretch group (*P all* > 0.05; Fig. 2a). Furthermore, In PAFs treated with vehicle lentivirus, stretch significantly increased the collagen I and III protein expression (*P all* < 0.05; Fig. 2b). However, there was no difference in collagen I and III protein expression between AAT1 group and AAT1 + stretch group (*P all* > 0.05; Fig. 2b). These results indicated that downregulation of endogenous SO_2_/AAT1 pathway might be an important pathogenesis of mechanical stretch-induced excessive collagen expression in PAFs.

### Endogenous SO_2_/AAT1 pathway inhibited stretch-induced TGF-β1/Smad2/3 pathway in PAFs

To explore the mechanism by which endogenous SO_2_ inhibited stretch-induced excessive collagen expression in PAFs, we compared stretch-induced TGF-β1 expression and phosphorylation of Smad2/3 with or without AAT1 overexpression treatment. Compared with vehicle group, TGF-β1 expression and phosphorylation of Smad2/3 in PAFs of vehicle + stretch group were markedly increased (*P all* < 0.05; Fig. 2c), while, as compared with vehicle + stretch group, TGF-β1 expression and phosphorylation of Smad2/3 in PAFs of AAT1 + stretch group were obviously decreased (*P all* < 0.05; Fig. 2c). However, there was no difference in TGF-β1 expression and phosphorylation of Smad2/3 between AAT1 and AAT1 + stretch group (*P all* > 0.05; Fig. 2c). The results suggested that TGF-β1/Smad2/3 pathway might be involved in the mechanisms by which SO_2_/AAT1 pathway regulated the stretch-induced excessive collagen expression in PAFs.

### Downregulation of endogenous SO_2_/AAT1 pathway induced excessive collagen expression in PAFs

**Figure 3:**
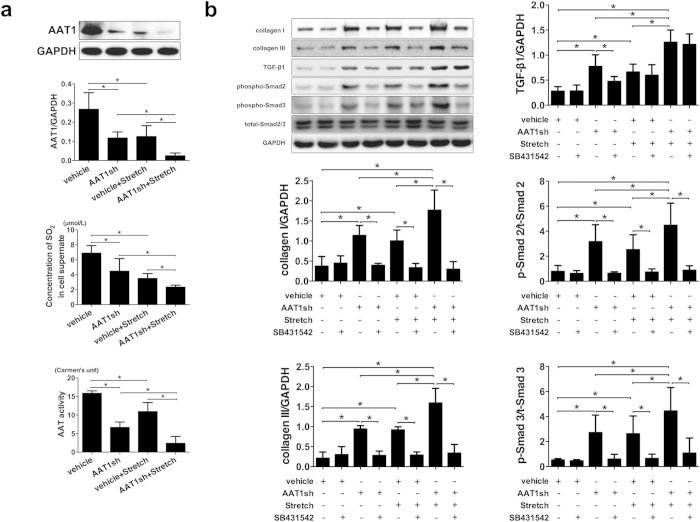
Knockdown of AAT1 augmented collagen remodeling and activated the TGF-β1/Smad2/3 pathway in PAFs. (**a**) The effect of AAT1 knockdown on the SO_2_/AAT1 pathway. (**b**) The effect of AAT1 knockdown and SB431542 on collagen remodeling and the TGF-β1/Smad2/3 pathway. *P < 0.05, data are presented as mean ± SD (n = 5) and ANOVA followed by *post-hoc* analysis was used. AAT1sh: AAT1 knockdown. SB431542: a TGF-β1/Smad2/3 inhibitor, concentration of 5 μmol/L.

To further verify the role of endogenous SO_2_/AAT1 pathway in the regulation of collagen protein expression, we downregulated endogenous SO_2_ levels by transfecting lentivirus vector containing AAT1 shRNA into primary PAFs. The data showed that AAT1 knockdown inhibited AAT1 protein expression and AAT activity in the PAFs and decreased SO_2_ content in the cell supernatant (*P all* < 0.05; Fig. 3a), which mimicked the effect of stretch on the endogenous SO_2_/AAT1 pathway in PAFs. Moreover, AAT1 knockdown induced excessive collagen I and III expressions in PAFs (*P both* < 0.05; Fig. 3b), which also mimicked stretch-induced excessive collagen protein expression in PAFs. These results further supported that deficiency of endogenous SO_2_/AAT1 pathway played an important role in stretch-induced excessive collagen expression in PAFs.

### Downregulation of endogenous SO_2_/AAT1 pathway activated the TGF-β1/Smad2/3 pathway in PAFs

Furthermore, we explored the role of endogenous SO_2_/AAT1 pathway in the regulation of TGF-β1/Smad2/3 pathway by knocking down AAT1 expression in PAFs. In accordance with the change of collagen in PAFs, TGF-β1 expression was increased and phosphorylation of Smad2 and Smad3 was activated by AAT1 knocking down (*P all* < 0.05; Fig. 3b), which mimicked stretch-activated TGF-β1/Smad2/3 pathway. Still, SB431542, a TGF-β1/Smad2/3 pathway inhibitor, was used to manipulate TGF-β1 pathway. The western blot results showed that SB431542 markedly inhibited phosphorylation of Smad2 and Smad3 in PAFs either in AAT1 knockdown group, stretch group or in AAT1 knockdown + stretch group (*P all* < 0.05; Fig. 3b). More importantly, AAT1 knockdown-induced increase in collagen I and III expressions were reversed by SB431542 (*P both* < 0.05; Fig. 3b). The blocking effect of SB431542 was also present in stretched PAFs and AAT1 knockdown + stretched PAFs (*P both* < 0.05; Fig. 3b). These results further supported that TGF-β1/Smad2/3 pathway mediated the inhibitory effect of endogenous SO_2_/AAT1 pathway on the stretch-induced excessive collagen expression.

### Deficiency of SO_2_/AAT1 pathway was involved in stretch-induced collagen accumulation *in vivo*

**Figure 4:**
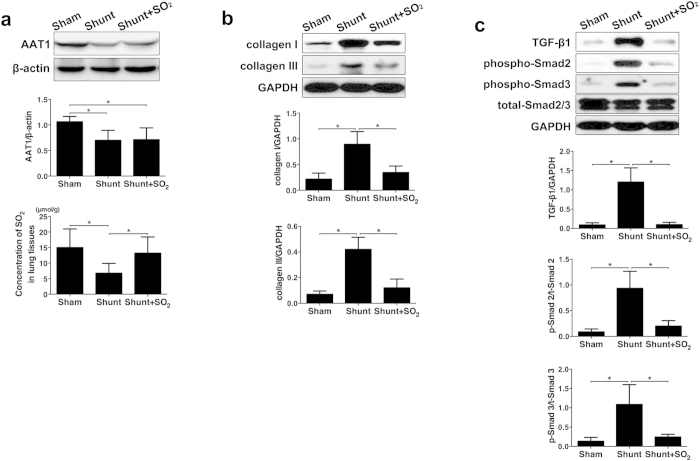
The level of SO_2_/AAT1 pathway and the expression of collagen and TGF-β1/Smad2/3 pathway in rats. (**a**) The expression of AAT1 and the concentration of SO_2_. (**b**) The effect of SO_2_ derivatives on collagen remodeling. (**c**) The effect of SO_2_ derivatives on the TGF-β1/Smad2/3 pathway. *P < 0.05, data are presented as mean ± SD (n = 8) and ANOVA followed by *post-hoc* analysis was used.

To validate the effect of endogenous SO_2_ on the stretch-induced excessive collagen expression *in vivo*, a rat model of high pulmonary blood flow-induced pulmonary vascular collagen accumulation was used. The data showed that compared with those of sham group, AAT1 expression and SO_2_ content were decreased in the lung tissue of rats in shunt group (*P both* < 0.05; Fig. 4a), while collagen I and III expression and TGF-β1 expression were upregulated, and phosphorylation of Smad2 and Smad3 was activated (*P all* < 0.05; Figs. 4b and 4c). However, compared with shunt group, SO_2_ content was increased in the lung tissue of rats in shunt + SO_2_ group (*P* < 0.05; Fig. 4a), while collagen I and III expression and TGF-β1 expression were decreased, and phosphorylation of Smad2 and Smad3 was inhibited (*P all* < 0.05; Fig. 4b-4c). The above data from the *in vivo* study also suggested that endogenous SO_2_/AAT1 pathway was involved in stretch-induced collagen accumulation, possibly via TGF-β1/Smad2/3 pathway.

## Discussion

Pulmonary hypertension, caused by a variety of underlying diseases, ultimately leads to right heart failure^21^. The excessive collagen deposition in the pulmonary artery is one of the important pathologic elements of pulmonary hypertension and pulmonary vascular structural remodeling^22^,^23^. Adventitial fibroblasts are classically defined as the cells that generate collagen, are considered to be the primary source of most extracelluar matrix components^24^,^25^,^26^, and sense cyclic stretch resulted from pulsatile blood flow. During the development of pulmonary hypertension, exacerbated mechanical stretch could be sensed by fibroblasts in the vessel wall and promote vascular structural remodeling by stimulating abnormal extracelluar matrix accumulation. To date, a growing number of studies have demonstrated the relationship between mechanical stretch and collagen synthesis. In 1976, Leung *et al.* found that cyclic stretching resulted in an increased rate of synthesis of collagen I and collagen III in arterial smooth muscle cells of rats^27^. In stretched cardiac fibroblasts, an increase in both collagen I and collagen III mRNA expression and total procollagen levels was also found^28^,^29^. However, how the mechanical stretch could induce excessive collagen expression was unclear.

Recently, endogenous SO_2_ , a new gaseous signal molecule, attracts more and more attention in the field. In cardiovascular system, SO_2_ is generated from sulfur-containing amino acid metabolism pathway via AAT transamination. Previous studies suggested that endogenous SO_2_ /AAT played an important role in maintaining the normal systemic and pulmonary vascular structure by inhibiting vascular smooth muscle cell proliferation, enhancing vascular smooth muscle cell apoptosis, and alleviating the inflammatory response of endothelial cells. Also, SO_2_ was reported to promote hypoxia-halted pulmonary vascular collagen degradation. However, whether endogenous SO_2_ is involved in the development of mechanical stretched-induced excessive collagen expression is unclear. In the present study, a mechanical stretch (60 cycles/min, 1 Hz, 20% elongation) for 24 hours was applied in rat primary PAFs to established stretch-induced cell model. In the mechanical stretch-treated PAFs, we firstly investigate if there was any change in endogenous SO_2_/AAT1 pathway. As expected, stretch stress destroyed endogenous SO_2_ production, presented by decreased AAT1 protein expression, lowered AAT activity and subsequently reduced SO_2_ content in cell culture supernatant. On the contrary, stretch stress induced a significant increase in collagen I and III expression. The data suggested that SO_2_/AAT1 pathway might be involved in the stretch-induced excessive collagen deposition.

To further explore the role of deficiency of SO_2_/AAT1 pathway in the development of excessive collagen expression induced by mechanical stretch, we manipulated the level of SO_2_/AAT1 pathway via overexpression or knockdown of AAT1. Firstly, we overexpressed AAT1 in PAFs with recombinant lentivirus to antagonize stretch-induced reduction of endogenous SO_2_ production. We found that in vehicle group, stretch stress induced marked collagen I and III accumulation, but it could not induce any increase in collagen I and III in PAFs overexpressing AAT1. Secondly, we downregulated SO_2_/AAT1 pathway by AAT1 knockdown in PAFs. The data showed that the effect of AAT1 knockdown on the collagen expression in static PAFs without stretch stress perfectly reappeared the effect of mechanical stretch on the collagen expression. These data provide evidence to suggest that sufficient SO_2_ could block stretch-induced collagen abnormal expression. However, the mechanisms by which SO_2_ inhibited stretch-induced excessive collagen expression attracted our further studies.

TGF-β1 pathway plays an important role in extracellular matrix metabolism. TGF-β1 binds to the transmembrane TGF-β receptors that have serine/threonine kinase activity. Activated TGF-β receptors induced a series of phosphorylation cascade and promoted phosphorylation of Smad2/3. Phosphorylated Smad2/3 formed a heteroligomeric complex with Smad4. The Smad complex is imported into the nucleus and regulates the expression of target gene transcription for ECM proteins including collagen^30^,^31^. In hamsters with cigarette smoke-induced pulmonary artery remodeling and pulmonary hypertension, TGF-β1, phospho-Smad2 and phospho-Smad3 were markedly increased^32^. Knockdown of Smad2/3 could restrain proliferation and migration of fibroblasts and reduced collagen I and collagen III expression. Therefore, we speculated that the TGF-β1/Smad2/3 pathway might be involved in the regulation of stretch-induced collagen expression by endogenous SO_2_. In our present study, the data showed that TGF-β1/Smad2/3 pathway in PAFs was activated by stretch stress, which could be prevented by AAT1 expression and exacerbated by AAT1 knockdown. Furthermore, we used a TGF-β1/Smad2/3 inhibitor, SB431542, which could inhibit the phosphorylation of Smad2/3. The data showed that treatment with SB431542 could eliminate the increase in collagen I and III protein expression induced by AAT1 knockdown, stretch or AAT1 knockdown plus stretch. These data suggested that TGF-β1/Smad2/3 pathway was involved in the mechanism by which endogenous SO_2_/AAT1 pathway inhibited stretch-induced excessive collagen accumulation.

In addition to the *in vitro* experiment, we further validated the role of SO_2_/AAT1 pathway in the stretch-induced collagen accumulation by studies *in vivo.* The experiment was performed in a rat model of high pulmonary blood flow-induced pulmonary vascular collagen remodeling. Our results showed that AAT1 expression and SO_2_ content in lung tissue of shunt rats with high pulmonary blood flow were markedly decreased, along with the increased expression of collagen I and III in lung tissues. While, SO_2_ derivatives inhibited collagen deposition, TGF-β1 expression and phosphorylation of Smad2/3 in the lung tissues of shunt rats. These data of the *in vivo* study further confirmed that SO_2_/AAT1 pathway played a pivot role in stretch-induced collagen accumulation.

In conclusion, our study for the first time suggested that mechanical stretch stress inhibited SO_2_/AAT1 pathway in PAFs, and disturbed SO_2_ production was an important pathogenesis of stretch-induced excessive collagen deposition *in vitro* and *in vivo*. Mechanistically, TGF-β1/Smad2/3 pathway was involved in the mechanisms by which endogenous SO_2_/AAT1 pathway protected against stretch-induced excessive collagen accumulation. These findings provided an in-depth insight of the pathogenesis of pulmonary vascular structural remodeling and indicated SO_2_/AAT pathway as a potential target for the treatment of pulmonary hypertension.

## Methods

### Ethics statement

Study protocols were approved by the Animal Research Ethics Committee of Peking University First Hospital (Permit Number: J201205). All experiments were performed in accordance with approved guidelines of Peking University First Hospital.

### Cell culture of primary pulmonary fibroblasts and treatment

Primary rat pulmonary artery fibroblasts (PAFs) were cultured using the method of tissue explant culture. SD rats weighing 150–180 g were intraperitoneally injected with 12% urethane (10 ml/kg). After anesthesia, the thoracic and abdominal skin were disinfected using 3% iodine tincture and 75% alcohol. The pulmonary was removed in the laminar flow bench rapidly and put into HPSS buffer (NaCl 130 mM, KCl 5 mM, CaCl_2_ 1.5 mM, MgCl_2_ 1.5 mM, HEPES 10 mM, and Glucose 10 mM). The tunica intima and tunica media were then removed before cutting the tunica adventitia into about 2 mm^2^ pieces. The tunica adventitia was attached to the Petri dishes evenly before being immersed in Dulbecco’s Modified Eagle Medium/F12 (DMEM/F12, Invitrogen, Carlsbad, USA) containing 20% fetal bovine serum (FBS, Invitrogen, Carlsbad, USA), 100 U/ml penicillin and 100 μg/ml streptomycin (Invitrogen, Carlsbad, USA). The Petri dishes were cultured in an incubator at a temperature of 37 °C, with 5% CO_2_. After the PAFs reached around 80% confluence, they were passaged and the medium was replaced with fresh DMEM/F12 containing 10% FBS. The standard for the identification of rat PAFs was positive vimentin, negative smooth muscle actin and the von Willebrand factor. SB431542 (Selleck Chemicals, Houston, USA), a TGF-β1/Smad2/3 inhibitor, was added to the cell culture supernatant 1 hour before mechanical stretching at a concentration of 5 μmol/L.

### Mechanical stretch stimulation

The isolated PAFs were inoculated with a density of 5 × 10^6^ per well in silicone elastomer-bottomed and pronectin-coated plates (Flexcell International Corp. McKeesport, PA, USA). Synchronization medium absent of FBS was incubated with cells for 24 hours before mechanical stretching. On the following day, the medium was replaced with fresh DMEM/F12 containing 10% FBS. A computer-controlled cyclic strain unit (FX-5000, Flexcell International Corp. McKeesport, PA, USA) was used to subject PAFs to mechanical stretching (60 cycles/min, 1 Hz, 20% elongation) for 24 hours under conditions of 37 °C in a 5% CO_2_ atmosphere. The static (non-stretched) cells were seeded in ordinary 6-well plates with the same density.

### Overexpression and knockdown of AAT1 in PAFs

A lentivirus vector containing complementary DNA (cDNA) encoding AAT1 (Vigene Bioscience, Jinan, China) was used to overexpress AAT1. A lentivirus vector containing small hairpin RNA (shRNA) targeting AAT1 (Cyagen Bioscience, Guangzhou, China) was used to knock down AAT1. Primary rat PAFs were seeded in culture flasks of 25 cm^2^ before virus transfection. When the cell confluence achieved around 50%, both of the recombinant lentiviruses as well as the lentivirus vector (vehicle) were infected separately for 48 hours at a multiplicity of infection (MOI) of 20 used by polybrene according to the manufacturer’s instructions. Puromycin was used to select the stable expression cells. The lowest concentration (4 μg/ml) that killed 100% of non-transfected PAFs in 3–4 days from the commencement of selection was chosen as the optimal concentration. Fresh puromycin-containing growth medium was exchanged every 3 days. After continuous selection with puromycin for approximately 3 weeks, the surviving cells were used for the following experiments. The efficiency of overexpression and knockdown was detected by western blot analysis.

### Western blot analysis

After the 24-hour stretch, PAFs were washed with phosphate buffered saline (PBS, 0.1 mol/L, pH 7–7.4) three times, lysed in lysate buffer at 4 °C for 30 minutes, and then scraped off with a cell scraper before being mixed with a quarter volume of loading buffer (5×). After 8 weeks, rat lung tissue homogenate was centrifuged at 12000 rpm for 30 minutes and the supernatant of each sample was mixed with equal volumes of loading buffer (2×). All the cell protein mixture and rat lung tissue protein mixture were boiled in 100 °C water and cooled down to room temperature. The concentration of protein was detected using a Bradford protein assay kit (M173-KIT, Amresco, America). Equal amounts of protein (50 μg) were separated on 10% SDS-polyacrylamide gels and transferred to nitrocellulose membranes. Strips were blocked with 5% nonfat blocking milk for 1 hour at room temperature. Strips were incubated overnight with primary antibodies against AAT1, collagen I, collagen III, TGF-β1, phospho-Smad2, phospho-Smad3, total-Smad2/3, and GAPDH which were diluted in PBS with 0.05% Tween 20 (TBST) at 4 °C. The strips were rinsed by TBST for 30 minutes, and then incubated with secondary antibodies for 1 hour at room temperature. All the protein bands were detected by ECL Western Blotting Detection Reagents (Amersham, GE Healthcare, UK) and analyzed by the Biological Electrophoresis Image Analysis System.

### High-performance liquid chromatography (HPLC) for measurement of SO_2_ production in PAFs and rat lung tissues

The concentration of SO_2_ in the cell culture supernatant and rat lung tissues was determined by high-performance liquid chromatography (HPLC; Agilent 1100 series, Agilent Technologies, CA, USA). At the end of the mechanical stretching experiment, the culture supernatant (100 μl) was aspirated into a clean tube. After 8 weeks, rat lung tissue homogenate was centrifuged at 12000 rpm for 30 minutes, and the supernatant (100 μl) was aspirated into a clean tube. 70 μl of 0.212 M sodium borohydride in 0.05M Tris-HCL (pH 8.5) was added to the supernatant and the mixture was incubated at room temperature for 30 minutes. 5 μl of 70 mM monobromobinane in acetonitrile was then added into the mixture and incubated at 42 °C for 10 minutes. Afterwards, 40 μl of 1.5M perchloric acid was added followed by vortex mixing. The mixtures were centrifuged at 12400 g for 10 minutes to remove the protein precipitates. The supernatant was mixed with 10 μl of 2.0 M Tris-HCL for neutralization, gently mixed, then centrifuged again at 12400 g for 10 minutes. All centrifugation steps were at room temperature. The final supernatant (100 μl) was transferred into foil-wrapped tubes and 10 μl of the supernatant was injected into a HPLC column. The column was equilibrated with buffer composed by methanol, acetic acid, and water in the ratio of 5.00: 0.25: 94.75 (by volume, pH 3.4). Sulfite-bimane adduct was detected by excitation at 392 nm and emission at 479 nm.

### Determination of AAT activity in PAFs

AAT activity of PAFs was detected using an AAT Activity Determination Kit (Nanjing Jiancheng Biological Engineer Academy, Nanjing, China) by colorimetry assay. At the end of the mechanical stretching experiment, the collected cells in each well were washed with PBS 3 times. The samples were then centrifuged at 1000 rpm/min for 10 minutes at room temperature. The supernatant was decanted and the precipitate was homogenated with 0.1 mol/L PBS that was used to detect AAT activity using the Reitman-Frankel method.

### Animal preparation and grouping

Twenty-four Male Sprague Dawley rats (provided by the Animal Research Committee of the First Hospital, Peking University) weighing from 130 to 180 g were divided randomly into three groups for treatment (n = 8 each group): the sham group, the shunt group and the shunt + SO_2_ group. Sham rats were treated with physiological saline (0.1 ml/kg body weight) via intravenous injection; rats in the shunt group and the shunt + SO_2_ group were subjected to an abdominal aorta-inferior vena cava shunting surgery to create an animal model of high pulmonary blood flow. Starting from the second day of the shunting surgery, rats from the shunt + SO_2_ group were given intraperitoneal injection of SO_2_ derivatives Na_2_SO_3_/NaHSO_3_ at a dosage of 0.54 mmol/kg: 0.18 mmol/kg body weight (molar ratio 3:1) for eight weeks^16^,^33^. Na_2_SO_3_/NaHSO_3_ was freshly dissolved in physiological saline (0.9%) before injection. All groups of rats were raised under identical conditions. The protein expression of AAT1, collagen I, collagen III and the TGF-β1/Smad2/3 pathway in lung tissues was detected by western blot. The concentration of SO_2_ in lung tissues was determined by HPLC.

### Statistical analysis

All the data are described using mean ± standard deviation. A two-sample independent t test was used to compare the differences between two groups. One-way analysis of variance (ANOVA) followed by *post-hoc* analysis (Newman-Keuls test) was used to compare the differences among three or more groups. All statistical analyses were performed using SPSS version 17.0 (SPSS, Chicago, IL, USA). A value of less than 0.05 was considered statistically significant in all analyses.

## Additional Information

**How to cite this article**: Liu, J. *et al.* Mechanical stretching stimulates collagen synthesis via down-regulating SO_2_/AAT1 pathway. *Sci. Rep.* **6**, 21112; doi: 10.1038/srep21112 (2016).
